# Spice Intake Among Chronic Gastritis Patients and Its Relationship With Blood Lipid Levels in South India

**DOI:** 10.7759/cureus.33112

**Published:** 2022-12-29

**Authors:** Tejaswi Nagireddi, Venkatashiva Reddy B, Siva Santosh Kumar Pentapati, Sai Subhakar Desu, Rajeev Aravindakshan, Arti Gupta

**Affiliations:** 1 Community and Family Medicine, All India Institute of Medical Sciences, Mangalagiri, Guntur, IND

**Keywords:** spicy, dyslipidemia, spice, diet, chronic gastritis

## Abstract

Introduction: Chronic gastritis is one of the most prevalent disorders affecting individuals. It affects hundreds of millions of people in different ways around the world. The objective of the study was to estimate the spice intake and its relationship with the blood lipid level among patients with chronic gastritis in the outpatient department of tertiary care hospital.

Methodology: The study design was a hospital-based cross-sectional study that was done in the Guntur district of Andhra Pradesh. The study population included 208 chronic gastritis patients between 20 and 60 years of age selected by systematic sampling. Detailed information on sociodemographic and lifestyle factors was collected using a questionnaire. Individual dietary intake data were collected by the detailed 24-hour dietary recall. Spice intake was calculated using Diet Calc Software. An independent t-test was used as a test for significance. The correlation was used to study the relationship between spicy food intake and dyslipidemia. P-value <0.05 was significant.

Results: A total of 208 patients were enrolled in the study with a response rate of 91%. The mean age of the studied patients was 45.15 ± 9.27 years, with 46.6% males and 53.4% females. Almost half (45.7%) of the participants had “mild” spicy food in their diet and almost two-fifths (39.9%) of participants had a “moderate or middle” degree of spice in their food. The mean dietary intake of condiments and spices by the participants was 34.19 (±22.18) gm/day. The current study showed higher spice intake was significantly correlated with impaired lipid profile levels with Kendall's tau_b correlation coefficient of 0.17 (p=0.01).

Conclusion: Because of the excessive use of spices in the Guntur region of Andhra Pradesh, people have grown accustomed to eating spicy food since childhood and therefore is at a higher risk of developing chronic gastritis, and dyslipidemia.

## Introduction

One of the most prevalent and stealth disorders affecting individuals is chronic gastritis (CG), which affects hundreds of millions of people in different ways around the world [1]. Gastritis is inflammation of the lining of the stomach. CG, on the other hand, is still one of the most prevalent major pandemic illnesses, with deadly complications including peptic ulcers and stomach cancer. Currently, more than half of the world's population may be suffering from CG [2]. The onset may either be sudden (acute gastritis) or occur slowly over time (CG). Changes in dietary intake and lifestyle, over time, have been leading to such serious conditions.

But the main cause of this CG is an infection due to the Helicobacter pylori bacterium [3]. Dietary factors such as the intake of spicy food that is food in spices/condiments which irritate the mucosal lining of the stomach could lead to such conditions [1]. Despite the high prevalence of Helicobacter pylori bacterium, only about 1%-3% of people are infected by this worldwide. CG condition due to Helicobacter pylori infection might sometimes even lead to cancer and death, if persistent. [2]. This indicates that the inflammation is influenced by several other factors such as the co-existence of intestinal microbes, host genetic factors, and dietary habits. Chronic H. pylori infection-related disease outcomes are influenced by the interaction of host genetics, immunological response, bacterial virulence expression, diet, availability of micronutrients, and microbiome structure. The burden of CG is therefore compounded due to poor diets [4].

Few research has looked into dyslipidemia association with atrophic gastritis and intestinal metaplasia, both of which are precancerous lesions of the stomach. Stomachache and gastric distention, the most common symptoms of CG, are associated with irregular mealtimes, irregular meal portions, dining out, meats, fried meals, sour foods, sweets, snacks, and salty foods consumption [1]. Patients suffering from this condition usually present with abdominal pain in the epigastric region which might mostly be accompanied by a burning sensation and loss of appetite. Other symptoms include belching, bloating, nausea and vomiting. An unexplained weight loss is also observed due to loss of appetite. This condition could be clinically assessed through various tests which indicate the levels of different biomarkers such as serum lipid and triglyceride levels [5,6]. There is a lack of research in the Indian population on the relationship between the spicy food intake of CG patients and the role of lipids as a mediator of these associations. On extensive review of the literature, we were not able to find out any studies in India specific to the objective. Among the studies done around the world, most of the studies were done in the last five years except one study which was done in 2001. All were observational studies and most used a cross-sectional study design and only one used a case-control study design. Most of the study designs used self-developed questionnaires and only one study used an already-validated questionnaire. All the studies were conducted on patients except one study which was conducted on university students. The objectives of the present study were to estimate the spice intake among patients of CG in the outpatient department in tertiary care hospitals and to determine the relationship between the blood lipid level, and spice intake among patients of CG in the outpatient department in tertiary care hospitals.

## Materials and methods

A cross-sectional study was conducted in the outpatient department of the tertiary care hospital AIIMS Mangalagiri, Andhra Pradesh, India, in August-September 2022. Male and female patients from 20 to 60 years of age suffering from CG were enrolled. Inclusion criteria included histologically diagnosed cases of CG or endoscopically diagnosed cases of CG or the patient should have had symptoms of gastritis at least once a week for the last six months or the patient taking any antacid formulations once a week for the last six months. Exclusion criteria were patients who will not give consent, patients who are very sick, pregnant women, cancer and HIV-positive patients, and patients requiring surgical intervention. For the calculation of the sample size, the prevalence of spicy food taken among CG patients was taken to be 25.1% as reported by a study from China [1]. the final sample size came to be 208 after taking 6% precision due to logistic reasons and a 95% confidence level.

A systematic sampling technique was adopted to recruit patients. Detailed information on socio-demographic and lifestyle factors was collected using a questionnaire. The following variables were included in the current study: age, gender, occupation, education, marital status tobacco use, alcohol use, physical activity, history of non-communicable diseases, and treatment history. Information on current gastrointestinal symptoms (stomach-ache, gastric distention, hiccup, belching, acid reflux, heartburn, abdominal bloating, nausea, abdominal pain, borborygmus, vomiting, diarrhea, and early satiety, appetite, the quantity of food, and symptom-related triggers. Individual dietary intake data were collected by the detailed 24-hour dietary recall. In addition, self-perceived spicy flavor (no, mild, middle, heavy) eating behaviors and the frequency of spicy food intake (never, 1-2 d/week, 3-5 d/week, 6 or 7 d/week), respectively were measured. Spice intake and Food group-based dietary intake were calculated using Diet Calc Software. Blood pressure recorded on the right arm supported at heart level in the sitting position was measured using electronic sphygmomanometers. The body mass index (BMI) was estimated as body weight (kg) divided by the height squared (m^2^). Glucose metabolism including fasting blood glucose (FBG), and postprandial blood glucose (PBG), of Venous blood samples, was recorded. Lipid metabolic markers including total cholesterol, triglycerides (TG), high-density lipoprotein cholesterol (HDL-C), low-density lipoprotein cholesterol (LDL-C), and very low-density lipoprotein cholesterol (VLDL-C) assessed by Dimension X PAND Plus analyzer was recorded.

The term “spice” is defined in the U.S. Code of Federal Regulations for specific labeling requirements. “Spice” is defined under 21 CFR Sec. 101.22(2) [7]. Dyslipidemia was defined using the 2013 ACC/AHA Cholesterol Treatment Guidelines [8]. For the 10- year risk of a first atherosclerotic cardiovascular disease (ASCVD), an event estimation online calculator was used [9]. A random number was generated using the lottery method from 1 to 10. The selected nth-eligible patient was enrolled in the study. He/she was provided a participant information sheet that explains the objectives and procedure of the study and the rights of the participants. If the patient agrees to participate, written consent was taken from the participant. Another random number was generated using the lottery method from 1 to 10 to recruit further patients systematically. The next selected number was added to the initial n^th^ number to enroll the subsequent patient. The study participants were interviewed according to the interview schedule, 24-hour dietary recall followed by anthropometric measurement, and blood sampling. The interview schedule was piloted to evaluate grammar and wording. The survey was conducted until the final sample size was achieved. Ethical approval was received from the All India Institute of Medical Sciences, Institute Ethics Committee. All COVID-19 precautions were followed during the data collection.

Continuous variables were described as means ± SD and categorical variables were presented as proportions. An Independent t-test was used as a test for significance. The correlation coefficient was calculated to estimate the association of spicy food intake frequency and blood lipid level with CG. The statistical analyses of the data were performed using SPSS V.23.0 software package and dietcalc software and p<0.05 (two-tailed) was considered significant.

## Results

A total of 208 patients were enrolled in the study with a response rate of 91%. The mean age of the studied patients was 45.15±9.27 years. The minimum and maximum ages were 20 years and 60 years in which almost three-fourths (73.6%) of the population were above 40 years and only one-fourth (26.4%) of the population were less than 40 years. Nearly 53.4% were females. The per capita income of 208 participants' mean was Rs 6,876.44 ± 35,165.1 (US$ 83.25 ± 425.71) and the median was Rs 3,000 (IQR 3,000), i.e., US $36.32. Most participants (34.6%) came under upper middle socio-economic status (Rs 3,944/- to Rs 7,888/-) (Table 1).

**Table 1:** Distribution of participants based on sociodemographic factors(n=208)

Domain	Category	n	%
Age in years	≤40	55	26.4
	>40	153	73.6
Gender	Female	111	53.4
	Male	97	46.6
Education Status	Illiterate or less than 5	56	26.9
	5th pass	45	21.6
	10th pass	51	24.5
	12th pass	20	9.6
	Graduate &above	36	17.3
Occupation	Business/self/volunteer	34	16.3
	Labour/cooli/farmer	55	26.4
	Private job	24	11.5
	Government	9	4.3
	House wife /Not working/student/retired/others	86	41.3
Socioeconomic Status (BG Prasad)	Upper [INR 7889 & above]	17	8.2
	Upper Middle [INR 3944 - 7888]	72	34.6
	Middle [INR 2367 - 3943]	56	26.9
	Lower Middle [INR 1183 - 2366]	48	23.1
	Lower [INR Below 1183]	15	7.2
	Total	208	100.0

Table 2 shows almost (91.8%) of participants were not using tobacco with more than two-thirds (85.1%) of participants who do not consume alcohol. There are almost two-thirds (63.0%) of participants have the habit of brisk walking for 30 minutes five days per week.

**Table 2:** Distribution of participants based on behavior (n=208)

Variable	Category	n	Percent
Current Tobacco Use	No	191	91.8
	Yes	17	8.2
Current Alcohol Use	No	177	85.1
	Yes	31	14.9
Current 30 min brisk walking 5 days/week	No	131	63.0
	Yes	77	37.0

**Figure 1:**
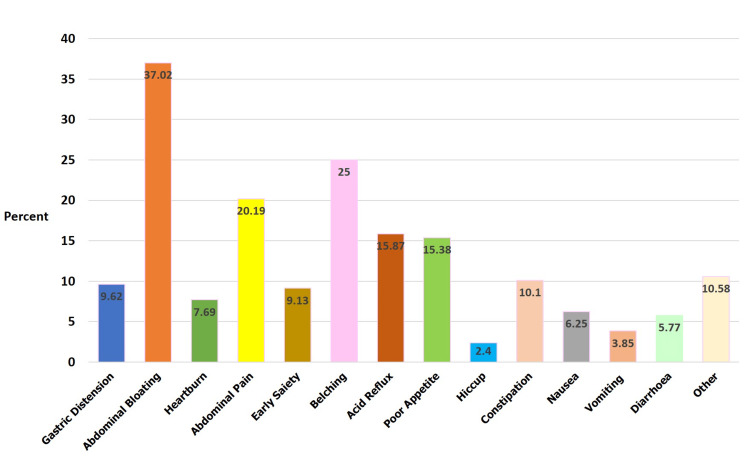
Distribution of participants based on gastritis symptoms (multiple options) (n=208)

Figure 1 shows almost two-fifths (37.02%) of participants with “abdominal bloating” as the presenting gastric symptom followed by one-fourth (25%) of participants with “belching” as the gastric symptom.

Table 3 shows almost half (45.7%) of the participants have “mild” spicy food in their diet and almost two-fifths (39.9%) of participants have a “moderate or middle” degree of spice in their food. The frequency of spicy food intake per week is equal for both “1-2 days/ week” and “3-5 days/ week” which is about (36.1%). Furthermore, almost more than three-fourths (82.7%) of participants have not stopped eating spicy foods. In the remaining (17.3%) of participants who have stopped having spicy foods, the mean “number of years since they stopped eating spicy food” is 3.04±4.31.

**Table 3:** Distribution of participants based on their behavioural patterns of spicy food intake (n=208)

Variable	Category	N	%
Behaviour of Spicy food intake in the diet	Heavy	24	11.5
	Middle	83	39.9
	Mild	95	45.7
	No	6	2.9
	Total	208	100.0
Frequency of eating spicy food	1-2 days/ week	75	36.1
	3-5 days/ week	75	36.1
	6 or 7 days/ week	48	23.1
	<1 day/ week	10	4.8
Stopped eating spicy food	No	172	82.7
	Yes	36	17.3
Years of stopped eating spicy food Mean (SD) (n=36)		3.04(4.31)	
Years of eating Spicy food before stopping Mean (SD) (n=36)		28.22(10.26)	

**Figure 2:**
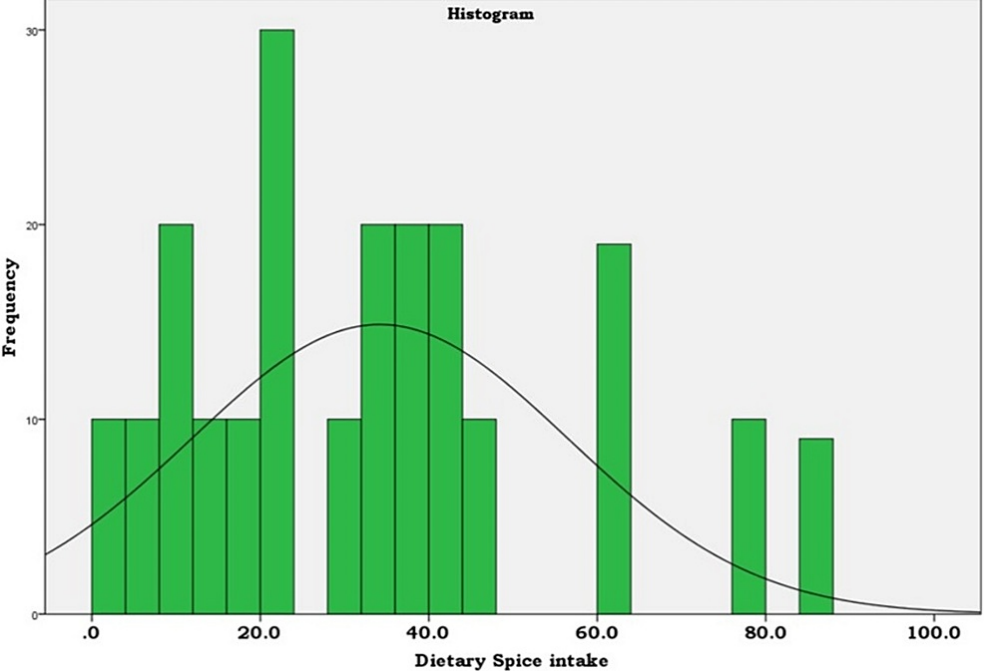
Histogram showing dietary intake of spices (g/day) among study participants

Figure 2 shows the mean dietary intake of condiments and spices by the participants (n=208) using 24-hour dietary recall was 34.19±22.18 g/day and the median was 35.00 g/day with IQR 24.1.

Table 4 shows the mean and standard deviation of spices and condiments in different domains. Males were having spicier foods with a mean (of 37.05± 22.98). Working participants were having spicier foods with a mean of 34.33 ± 22.61, There was higher spicy food intake in persons with “tobacco use” where the mean was 39.53 ± 27.10 and “alcohol use” where mean (34.13) and Std. Deviation (21.90). There was no significant association between the intake of spices and condiments in study participants based on different domains.

**Table 4:** Dietary intake of spices and condiments (g/day) among study participants by various factors *Reference 1

Variable	Category	N	Spices and condiments (gm/day)		t	df	CI Lower	CI Upper	P-value
			Mean	Std. Deviation					
Gender	Female*	111.00	31.69	21.26	-1.75	206.00	-11.41	0.69	0.08
	Male	97.00	37.05	22.98	-1.74	197.07	-11.44	0.73	0.08
Age in years	<=40*	55.00	34.47	21.51	0.11	206.00	-6.51	7.28	0.91
	>40	153.00	34.09	22.50	0.11	99.36	-6.41	7.17	0.91
Occupation	Working*	122.00	34.33	22.61	0.10	206.00	-5.85	6.50	0.92
	Not working	86.00	34.00	21.71	0.11	187.59	-5.81	6.46	0.92
Education status	10 standard or more*	107.00	35.56	21.15	0.92	206.00	-3.25	8.89	0.36
	Less than 10 standards	101.00	32.74	23.26	0.91	201.31	-3.27	8.91	0.36
Socioeconomic status	Middle class or higher*	145.00	35.29	22.51	1.08	206.00	-2.98	10.22	0.28
	Lower Middle or lower	63.00	31.67	21.40	1.10	123.63	-2.87	10.12	0.27
Nutrition status	Normal	20.00	29.00	21.16	-1.10	206.00	-16.03	4.54	0.27
	Overweight/Obesity	188.00	34.74	22.28	-1.15	23.71	-16.08	4.59	0.26
Comorbidities Present	No*	21.00	27.67	18.61	-1.42	206.00	-17.30	2.78	0.16
	Yes	187.00	34.93	22.48	-1.66	27.01	-16.25	1.73	0.11
Surgery is done in past	No*	103.00	36.69	22.98	1.61	206.00	-1.10	10.99	0.11
	Yes	105.00	31.74	21.20	1.61	203.97	-1.10	10.99	0.11
Drugs used for comorbidities	No*	40.00	29.20	24.11	-1.59	206.00	-13.85	1.49	0.11
	Yes	168.00	35.38	21.61	-1.49	54.88	-14.52	2.16	0.14
Dyslipidemia	Yes*	122	33.33	21.94	-0.66	206	-8.25	4.07	0.51
	No	86	35.42	22.59	-0.66	179.7	-8.29	4.11	0.51
Knowledge of FOPL	No*	25.00	37.00	22.95	0.67	206.00	-6.15	12.53	0.50
	Moderate	183.00	33.81	22.12	0.66	30.42	-6.75	13.14	0.52
Tobacco product used	No*	191.00	33.72	21.72	-1.04	206.00	-16.88	5.26	0.30
	Yes	17.00	39.53	27.10	-0.86	17.88	-20.02	8.39	0.40
Alcohol product used	No*	177.00	34.20	22.30	0.02	206.00	-8.46	8.61	0.99
	Yes	31.00	34.13	21.90	0.02	41.66	-8.56	8.70	0.99

Table 5 shows different parameters in which the mean TG is 179.38±114.90 mg/dL, and the mean total cholesterol is 199.65±44.49 mg/dL. The mean BMI is 28.94±5.12 kg/m^2^.

**Table 5:** Summary of biochemical and physiological parameters in the study population (n=208)

Variable	N	Mean	Median	SD	First Quartile	Third Quartile	IQR
TG (mg/dl)	208	179.38	152.00	114.90	106.00	207.50	101.50
HDL-C (mg/dl)	208	46.49	41.40	32.04	35.28	47.88	12.60
VLDL-C (mg/dl)	203	38.90	29.80	33.94	21.30	41.80	20.50
LDL-C (mg/dl)	208	122.20	116.10	39.30	96.43	148.13	51.70
Total cholesterol (mg/dl)	208	199.65	194.50	44.49	166.00	226.00	60.00
PPBS (mg/dl)	197	215.89	189.00	101.93	133.50	287.50	154.00
FBS (mg/dl)	207	151.88	127.00	66.96	100.00	187.00	87.00
SBP (mm/Hg)	208	133.55	130.50	19.11	120.00	143.75	23.75
DBP (mm/Hg)	208	83.70	82.00	12.76	76.00	90.75	14.75
BMI (kg/m2)	208	28.94	28.25	5.12	25.25	32.08	6.83

Table 6 shows the burden of dyslipidemia among study participants. There were 38 (18.3%) adults with ≥ 7.5 % 10 years ASCVD risk with LDL of 70 to 189 mg/dL, 72 (34.6%) adults having type 1 or type 2 diabetes with LDL of 70 to 189 mg/dL, finally, 12 (5.7%) adults are having primary LDL-C ≥ 190 mg/dL.

**Table 6:** Burden of Dyslipidemia among studied patients using 2013 ACC/AHA Cholesterol Treatment Guidelines

Criteria	n (%)
Adults with clinical established ASCVD	0(0)
Adults with primary LDL-C≥ 190 mg/dL	12(5.7)
Adults (40–75 years of age) with either type 1 or type 2 diabetes with LDL of 70 to 189 mg/dl	72(34.6)
Adults (40–75 years of age) with ≥ 7.5 % 10 years ASCVD risk with LDL of 70 to 189 mg/dl	38(18.2)

Table 7 shows the correlation between dyslipidemia, and spicy food intake, among study participants. A statistically significant positive correlation was found between dyslipidemia and spicy food Intake among study participants with Kendall's tau_b Correlation Coefficient of 0.17.

**Table 7:** Correlation of Dyslipidemia, and Spicy food Intake among study participants (n=208) * Statistically Significant (p <0.05)

			Dyslipidemia	Spicy food Intake
Kendall's tau_b	Dyslipidemia	Correlation Coefficient	1.000	.171^*^
		Sig. (2-tailed)	.	.014
		N	208	208
	Spicy food Intake	Correlation Coefficient	.171^*^	1.000
		Sig. (2-tailed)	.014	.
		N	208	208
		Sig. (2-tailed)	.884	.223
		N	208	208
		Sig. (2-tailed)	.013	.
		N	208	208

## Discussion

This is the first of its kind study in India to quantify dietary spice intake among CG patients between 20 and 60 years of age in a tertiary care hospital in South India. In the present study, almost half (45.7%) of the participants were having mild spicy food in their diet and almost two-fifths (39.9%) of the participants were having a moderate or middle degree of spice in their food. Almost more than three-fourths (82.7%) of participants have not stopped eating spicy foods among which around one-fourth (23.1%) consume spicy food more than 6-7 times per week. This depicts that a high frequency of spice intake is significantly associated with the occurrence of CG. The present results were comparable to a study conducted in China in 2020 in which about 25.10% of the participants reported that the symptoms appeared due to spicy food intake [1]. A study conducted in Ethiopia in 2021 also revealed that eating spicy food has significantly contributed to the occurrence of gastritis [10,11]. The results were also relevant to that of a case-control study performed in China in 2016 in which there was a significant correlation between spicy food intake and the occurrence of CG [12].

More than three-fourths of participants reported having eaten spicy foods. The mean dietary spice intake was 34.19±22.18 g/day using 24-hour dietary recall. In 2018, a study from China among people more than 65 years of age reported the IQR for spice intake was 0-16.7 [3]. This discrepancy reflects the demographic and geographic distribution of study participants and preferences for spicy food In India, Guntur, Andhra Pradesh is known as the “Chilli city” and is known across the world for its chili growers.

Other recent studies from China found of the proportion of participants consuming spicy food weekly was 99.7%) [13], and 12.3% [14], respectively. This highlights the role of spicy food preferences in different geographic areas. In line with other research [15,16], participants who consumed spicy food more frequently were more likely to be young, working, male, and of a high socioeconomic level. Nearly, 17.3% of the participants stopped consuming spicy food. The mean years of eating spicy food before stopping were 28.22 years among these participants. A similar study was conducted in Korea in 2001 which showed that high levels of dietary intake of spicy food increased the risk of occurrence of CG [17]. A comparable study was carried out in Malaysia in 2016 regarding gastritis and its relevance with dietary factors and in which it was found that the frequency of consumption of spicy food was the highest above all other factors [18]. A study reported male patients likely need to limit the consumption of spicy foods to control gastritis symptoms [19]. The current study found that people who consumed spicy food more frequently appeared to have greater BMI, in contrast to an earlier study that found people with high levels of spicy food consumption had lower BMI levels when compared with non-consumers [20]. This may be due to increased palatability and appetite for spicy and oily food. In Guntur, Andhra Pradesh, people are generally accustomed to eating spicy food since early childhood. They regularly favor spicy meals. As a result, Andhra-style recipes for food are typically spicier than those for other cuisines [21]. This emphasizes the need for early dietary counseling to reduce the chances of CG.

It is hard to establish cause-and-effect correlations between dietary factors and CG because of this study's cross-sectional approach. However, it is hypothesized that patients with CG may have altered their diet to receive therapy or to lessen gastritis-related symptoms. Overall, this study's findings support earlier research indicating that there is a substantial relationship between symptoms of gastritis and spicy food intake. As it is well known, CG is a multi-stage, progressive, and chronic inflammatory illness with a wide range of symptoms. More diverse treatment models should be investigated, incorporating patient self-management abilities like daily nutrition, rather than relying simply on pharmacological therapy.

The current study recorded a mean triglyceride level of 179.38 mg/dl, a mean HDL-C level of 46.49 mg/dL, and a mean VLDL-C to be 38.90 mg/dL. For LDL-C the mean is 122.2 mg/dL and for total cholesterol, the mean was found to be 199.65 mg/dL. According to the Adult Treatment Panel III (ATP III) for dyslipidemia, the standard levels per guideline [22], the present study recorded higher triglyceride levels, lower HDL-C levels, and greater LDL-C levels than required. This depicts a notable disturbance in the blood lipid profile in case of the occurrence of CG.

In comparison with the results of the current study to that of a similar study conducted in Ethiopia in 2022, the serum HDL-C levels were found to be higher in the present study [23]. This may be due to the difference in the study population as the study in Ethiopia involved patients who immediately tested positive for H. pylori. A comparative study between H. pylori eradicated and H. pylori positive groups in China in 2022 revealed significantly decreased HDL and increased LDL in both H. pylori eradicated and H. pylori positive groups which point out to the long-term effect of gastritis on lipid profile [24]. A similar comparative study carried out in Hyderabad, India showed that patients infected with H. pylori had a statistically significant increase in LDL cholesterol levels and a decrease in HDL cholesterol levels than the controls [25]. Another study carried out in Japan in 2019, regarding the eradication of H. pylori recorded a significant increase in HDL levels while LDL, total cholesterol, and triglycerides levels were not altered significantly [26]. These studies depict a remarkable alteration in lipid profile in patients suffering from CG.

The present study found a significant correlation between dietary intake of spicy food and dyslipidemia among patients with CG. Similarly, a cross-sectional study conducted to investigate associations between spicy food intake and serum lipids levels in the Chinese rural population reported spicy flavor and intake frequency were consistently associated with decreased TC and non-HDL-cholesterol levels but mildly associated with elevated TG levels [5]. Another study also documented that CG patients showed higher serum triglyceride concentrations than normal subjects [3]. These results indicate that the dietary pattern of CG patients may have a relation to a change in the serum lipid level.

Several theories have been put out, however, the fact that the exact mechanisms underlying the potential negative impact of eating spicy food on dyslipidemia have not yet been thoroughly explored. The results of the present investigation could have significant clinical and public health implications. Reduced consumption of spicy foods may be an effective dietary intervention for the prevention of dyslipidemia in both healthy populations and high-risk individuals, particularly in areas where the consumption of spicy foods is normally high, like Andhra Pradesh, India. However, confirming this possible clinical relevance will require more data from prospective and randomized trials.

Although the study objectives could be achieved to a major extent, they involved a few potential limitations. Firstly, even though the 24-hour dietary recall method improved the accuracy of the estimation of spice intake, the approach to assess it may not reflect the usual food intake status as it may vary each day. Secondly, the cross-sectional study design restricted the establishment of a temporal relationship between spicy food consumption with gastritis. Thirdly, the assessment of spicy food consumption in the current study was self-reported and subject to measurement error.

## Conclusions

Guntur district, Andhra Pradesh, is a high spice-consuming area which could be due to the fact that the people are habituated to eating spicy food since early childhood and this has led to a greater risk of occurrence of CG, dyslipidemia, and other medical ailments. The majority of the participants lack knowledge about the food they consume and hence education methods targeting dietary interventions are the need of the hour to create awareness among the people.
